# Pyriproxyfen, villain or good guy? A brief review

**DOI:** 10.20945/2359-4292-2024-0154

**Published:** 2024-11-06

**Authors:** Andressa Pereira Cabral, Fabrício Pereira dos Santos Maia, D’Angelo Carlo Magliano, Jones Bernardes Graceli, Paula Soares, Eduardo Andrés Rios Morris, Leandro Miranda-Alves

**Affiliations:** 1 Universidade Federal do Rio de Janeiro Instituto de Ciências Biomédicas Laboratório de Endocrinologia Experimental Rio de Janeiro RJ Brasil Laboratório de Endocrinologia Experimental (LEEx), Instituto de Ciências Biomédicas, Universidade Federal do Rio de Janeiro, Rio de Janeiro, RJ, Brasil; 2 Universidade Federal do Rio de Janeiro Instituto de Ciências Biomédicas Rio de Janeiro RJ Brasil Programa de Pós-graduação em Farmacologia e Química Medicinal, Instituto de Ciências Biomédicas, Universidade Federal do Rio de Janeiro, Rio de Janeiro, RJ, Brasil; 3 Universidade Federal Fluminense Centro de Morfologia e Metabolismo Niterói RJ Brasil Centro de Morfologia e Metabolismo, Universidade Federal Fluminense, Niterói, RJ, Brasil; 4 Universidade Federal do Espírito Santo Laboratório de Endocrinologia e Toxicologia Celular Departamento de Morfologia Espírito Santo ES Brasil Laboratório de Endocrinologia e Toxicologia Celular, Departamento de Morfologia, Universidade Federal do Espírito Santo, Espírito Santo, ES, Brasil; 5 Universidade do Porto Instituto de Investigação e Inovação em Saúde Grupo de Sinalização e Metabolismo Celular Porto Portugal Grupo de Sinalização e Metabolismo Celular, i3S – Instituto de Investigação e Inovação em Saúde, Universidade do Porto, Porto, Portugal; 6 Universidade Federal do Rio de Janeiro Programa de Pós-graduação em Endocrinologia Faculdade de Medicina Rio de Janeiro RJ Brasil Programa de Pós-graduação em Endocrinologia, Faculdade de Medicina, Universidade Federal do Rio de Janeiro, Rio de Janeiro, RJ, Brasil

**Keywords:** Pyriproxyfen, endocrine disruptor, toxic effects

## Abstract

Pyriproxyfen (PPF) acts as a juvenile growth regulator, interfering with normal metamorphosis and blocking the development of insects into adulthood. Although the World Health Organization (WHO) considers the use of PPF at a concentration of 0.01 mg/L as unlikely to pose health risks, recent studies have unveiled potential risks associated with PPF exposure to non-target organisms. Exposure to PPF disrupts insect development primarily by mimicking juvenile hormones; therefore, concerns linger over its impact on unintended species. Studies have highlighted the adverse effects of PPF on aquatic invertebrates, fish, and amphibians and revealed mortality and developmental abnormalities in non-target mosquito species exposed to PPF-treated water. Moreover, PPF may act as an endocrine disruptor, interfering with hormonal pathways crucial for growth, reproduction, and behavior in exposed organisms. Amphibians, for instance, display altered reproductive physiology and developmental abnormalities due to disruptions in endocrine signaling pathways caused by PPF. The ecological ramifications of PPF extend beyond direct toxicity to non-target species. Indirect effects include shifts in food web dynamics and ecosystem functioning. Reductions in insect populations, induced by PPF, can disrupt food availability for higher trophic levels, potentially destabilizing community structure and ecosystem equilibrium. Given mounting evidence of unintended consequences, robust risk assessment and regulatory oversight are imperative. Accurate classification of PPF by regulatory bodies is essential to balancing its role in disease control and pest management benefits with the need to safeguard non-target species and maintain ecosystem health. Future research must prioritize comprehensive assessments of PPF's ecological impact across various habitats and taxa to inform evidence-based policymaking.

## INTRODUCTION

**Figure 1:**
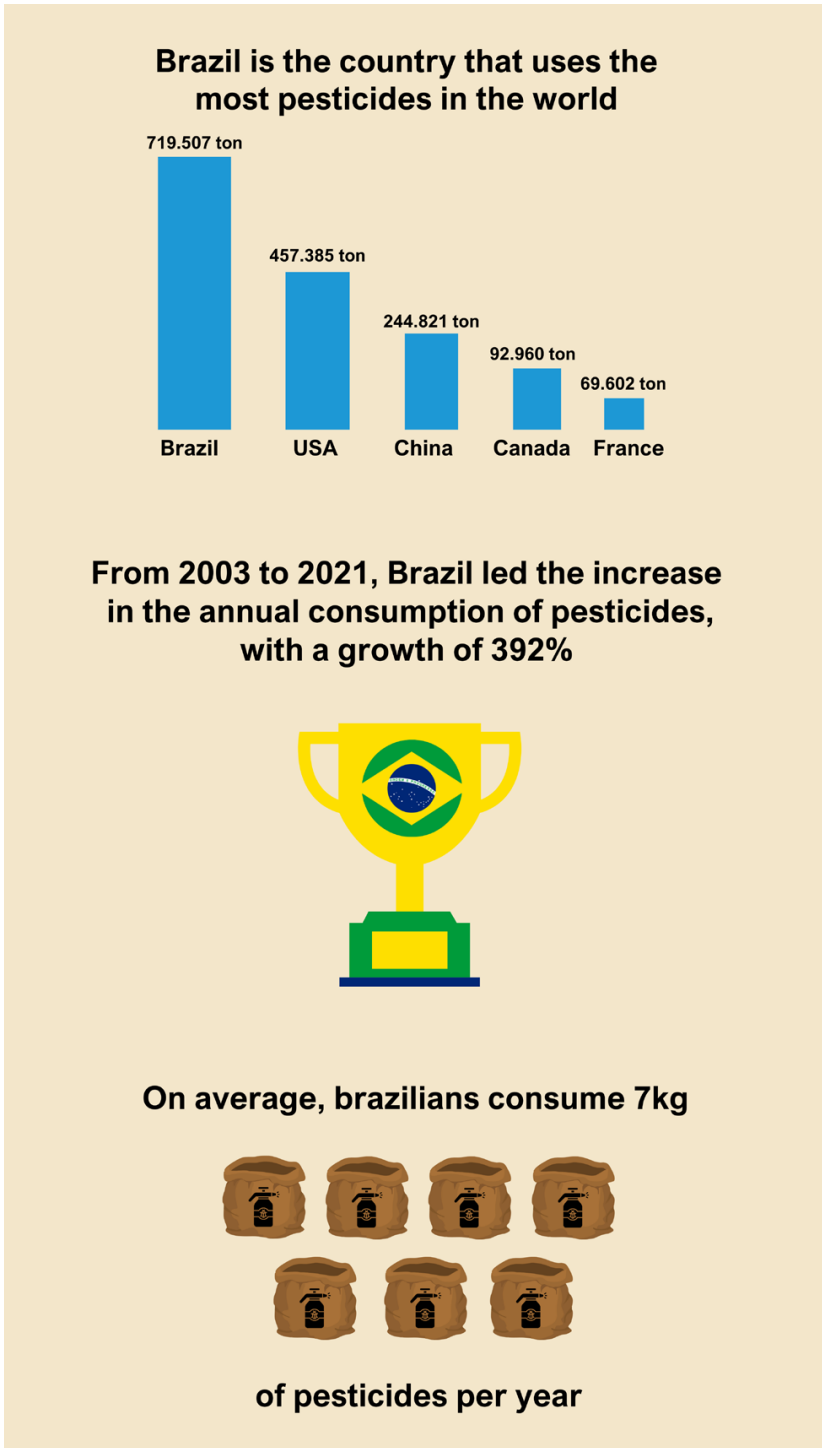
Statistics on pesticide exposure and consumption in Brazil. Figure created using Canva.

Vector-borne diseases have been a major public health challenge for many years, causing hundreds of thousands of deaths annually (1,2). Among the species capable of transmitting diseases such as dengue, yellow fever, chikungunya, and Zika, we find mosquitoes of the genus *Aedes aegypti* (3,4). With the main aim of inhibiting the proliferation of this mosquito and avoiding the spread of vector diseases, strategies have been developed to curb this common vector (5,6). The use of substances for chemical control (*e.g.*, insecticides) has been an alternative path to prevent the spread of vector-borne diseases (Figure 1) (7,8). These substances gained strength and popularization after the end of the Second World War and have been improved over the decades, mainly due to the development of mosquito resistance (9) and the toxicity these chemicals cause to human health and ecosystems (10,11).

Different classes of insecticides are commonly used today. Chemical compounds mimicking insect growth regulators (IGRs) have gained prominence in the last decades (12,13). Different from other classes, IGRs are very selective as analogs of juvenile hormone (JH) (14) and are specifically capable of interfering with physiological processes triggering metamorphosis in insects (15,16). Despite having no effective activity in causing death (12,13), its presence in the mosquito environment promotes developmental abnormalities, impairing the mosquito's normal transition to adult stages (3,13,14).

Several synthetic substances are part of the IGR group. The most known IGRs are methoxyfenozide, tebufenozide, and, mainly, pyriproxyfen (PPF) (14,15), which due to its high effectiveness, has gained space as the most used IGR (16,17).

Notably, PPF – 2-[1-methyl-2-(4-phenoxyphenoxy) ethoxy] pyridine – is an effective pesticide used to control the proliferation of several arthropods (18,19). During development, JH maintains larvae features, and during metamorphosis, JH levels decrease to allow the development of essential structures for future survival (20). As a JH analog, PPF inhibits metamorphosis and impairs the correct establishment of adult characteristics such as wings, reproductive organs’ maturation, and external genitalia (20,21). Also, during the reproductive stages (20), JH plays a role in sexual behavior, contributing to pheromonal synthesis and vitellogenin expression, which is essential for the oocyte supply (22). Thus, PPF is capable of controlling the proliferation of mosquitoes and significantly reducing the vector-borne disease spread rate (20-22).

The World Health Organization (WHO, 2001) classifies PPF as unlikely to cause damage to health when used at a concentration of 0.01 mg/L (23). At this concentration, PPF is considered non-genotoxic and non-carcinogenic when used in its granule formulation. In addition, the Guidelines for Drinking Water Quality (GDWQ) International Program on Chemical Safety (IPCS) considered PPF safe for use in the control of the *Aedes aegypti* vector, including in drinking water for human consumption (24). However, over the last years, it has been observed that PPF is often used indiscriminately, which compromises its safe dose. Furthermore, results from some studies lead us to believe that there is a possible relationship between PPF and some histofunctional health disorders in non-target living beings (25-29).

**Figure 2:**
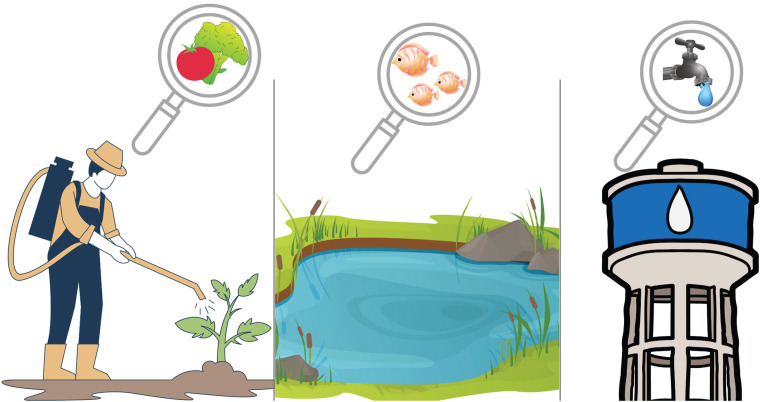
Possible routes of pesticide contamination and contact. Figure created using Canva.

The discussion about PPF as a potential persistent substance in the environment is relevant. Even though PPF may degrade over a short period of time (30-32), a recent study demonstrated that PPF could take long periods to degrade on soil surface. When sprayed on soil, PPF behaves like a translaminar insecticide (33) (Figure 2) and is able to cross the plant surface. Therefore, its half-life in crops can vary, reaching up to 3 weeks (33), and in some cases (*e.g.*, silty or salty loam soil), its half-life can reach 21 weeks (33). On water surface, PPF is susceptible to photodegradation and biological catalysis, reaching a half-life of 21 days, but in anaerobic conditions or sediments, its half-life can reach 750 days (32,33).

It has been observed that PPF bioaccumulates in living organisms (34,35), a finding that has to be better understood since PPF has been found in high concentrations in liver, fat, kidney, and blood of rats after oral administration (34,35). This means that PPF's permanence in the environment could result in various consequences for non-target organisms. Studies have demonstrated that PPF in underwater ecosystems could cause developmental abnormalities in fish (35-37) and amphibians (38,39), and impair sex determination in crustaceans (40,41).

The effect of PPF on human health has not been studied yet, even when PPF is diluted directly in drinking water containers to control mosquito proliferation. In February 2016, the Brazilian Association of Public Health (Abrasco) published a technical statement correlating the utilization of PPF to control the Zika virus outbreak and the rise of microcephalic events in newborns in northern Brazil (42). Even though microcephalic events in newborns due to Zika virus infection were described later (43,44), the statement was controversial enough to bring PPF to discussion, but no consensus has been reached yet about the effects of PPF exposure in humans (43,44).

Due to evidence showing that PPF could produce effects in non-target organisms (45-48), the lack of control from chemical protection agencies in developing countries, and the absence of studies on its effects in humans, it is necessary to elucidate carefully the possible effects of PPF on the environment and collective health. Here, we collect some evidence about the effects of PPF exposure on different living organisms in terms of toxicity, endocrine system morphofunctionality, and consequences in health physiology.

## METHODS

This literature review aimed to investigate the effects of PPF on non-target organisms. A search was conducted using the keywords "pyriproxyfen," "non-target organisms," "ecotoxicology," "insect growth regulator," "environmental impact," and "aquatic toxicity." This literature search was performed across several major scientific databases, including PubMed, Scopus, Web of Science, and Google Scholar. Inclusion criteria focused on studies published in the last 8 years (2015-2023) that specifically examined the effects of PPF on non-target organisms such as beneficial insects, aquatic organisms, and other vertebrates and invertebrates. Articles presenting experimental data, systematic reviews, and meta-analyses were included. Exclusion criteria ruled out studies lacking relevant experimental data, non-systematic reviews, and articles that focused exclusively on target organisms (*e.g.*, mosquitoes) or did not primarily address PPF. An exception was made for one pivotal study published in 1999, as it provided significant insights into the early understanding of PPF's impact on non-target organisms and was the only relevant document available before 2016.

The selection process involved a two-stage screening. Initially, titles and abstracts were reviewed to assess relevance to the topic. Subsequently, potentially relevant articles were read in full to confirm their inclusion in the review. Data extracted from the selected studies were organized and analyzed to identify patterns, research gaps, and the potential impact of PPF on various groups of non-target organisms.

### Effects of pyriproxyfen exposure

#### Toxicodynamics

Notably, PPF is used to control populations of arthropods, mainly mosquito vectors of arboviruses, and is applied to crops, drinking water containers, and indoor environments (4,13). Due to its application in different spaces, PPF could reach some non-target organisms such as bees, aquatic life, mammals, and even humans (10,11,24,26,37). In the absence of evidence analyzing the effects of PPF on humans, most studies have been conducted to understand the possible consequences of PPF exposure on different *in vivo* models. Several of these studies suggest that PPF can cause harmful effects (Table 1) depending on its administration form, treatment duration, concentration, and the model itself (34-40).

**Table 1:** Main toxicological effects of pesticides in some vertebrate species

Model	Tissues analyzed	Exposure	Main results	Reference
Bee larvae	Cuticles, thorax, head, eyes	Topical treatment (within comb cells) with 4′-OH-pyriproxyfen (a pyriproxyfen metabolite) 1 µg in 1 µL of acetone daily for 10 days.	Changes in pigmentation and cuticular sclerotization, impaired development leading to death, abnormalities in the developmental process, and decreased growth.	32
C57BL/6 wild-type, 8-week-old male mice	Subventricular zones of the brain (neutrosphere culture and analysis)	4’-OH-pyriproxyfen (10^−2^ mg/L, 10^−1^ mg/L, or 30^−1^ mg/L) added to the proliferation medium for primary neurosphere cultures. The medium was renewed every 2 days for 7 days, and primary neurospheres were obtained.	Decreased thyroid hormone signaling, increased cell proliferation, dysregulation of genes related to neurogliogenesis, elevated Msi1 mRNA levels, and increased apoptosis, leading to disruptions in neurogenic processes.	49
*Xenopus tadpoles*	Brain	Exposure for 72 hours to 4’-OH-pyriproxyfen (10^−5^ mg/L, 10^−4^ mg/L, 10^−2^ mg/L, 10^−1^ mg/L, and 30^−1^ mg/L) added to the water, with daily renewal of the solution.	Delayed development, disrupted thyroid signaling leading to microcephaly, blocked T3 binding.	49
Pregnant mice	Body weight (of mothers)	Exposure to pyriproxyfen (30 mg/kg, 10^2^ mg/kg, 3×10^2^ mg/kg, and 10^3^ mg/kg), administered orally between gestation days 7 and 17.	Reduced weight in treated mothers, histological changes in puppies’ organs (including kidney, liver, heart, and brain), and fetal death at the highest concentration.	50
8-week-old male mice	Testes and body weight	Exposure for 28 days to oral pyriproxyfen (daily doses of 1200 mg/kg, 600 mg/kg, 320 mg/kg, 200 mg/kg, 100 mg/kg, 40 mg/kg, 20 mg/kg, 0 mg/kg).	Reduced body weight, shrinkage, and displacement of seminiferous tubules, reduction in seminiferous tubules and lumen diameter.	50
*Ceraeochrysa claveri* newly hatched (0-12 hours) larvae	Midgut and body fat	During the larval stage, oral feeding with *Diatraea saccharalis* egg clusters treated with pyriproxyfen 50 mg/L and 100 mg/L).	Increased intercellular spaces, decreased lipid droplets, vacuolization of trophocytes, significant mitochondrial damage, imbalanced youth hormone levels, suppression of embryogenesis, changes in metamorphosis and adult formation, and increased number of cytoplasmic granules.	51
Chicken embryos	Head and brain	Exposure to pyriproxyfen 0.01 mg/L (the maximum concentration allowed in drinking water) and 10 mg/L (simulation of high exposure) in a 50 μL solution, administered through a small opening in the eggshell, from 24 hours of incubation until embryonic day 10.	Reduced brain mass, changes in forebrain and midbrain morphologies, reduced cell proliferation and increased cell death in the brain, neurodevelopmental disorder, disturbed ossification and chondrogenesis processes.	52
Adult *Lithobates catesbeianus* (Bullfrog)	Adipose tissue	Exposure for 50 days to pyriproxyfen 0.002 g/L and 0.02 g/L in chlorine-free water in a tank, with both water and pesticide being renewed every 48 hours to maintain consistent toxicity levels.	Increased cumulative mortality during the prepupal to pupal stage (from 47.3% to 61.8%), delayed larval development, increased accumulation of abdominal body fat, changes in intestinal epithelium, stretching of intercellular spaces, and decreased brain mass.	53
*Odontophrynus americanus* (tadpoles in the pre-metamorphic stage)	Whole body	Exposure for 22 days to pyriproxyfen 0.01 mg/L and 0.1 mg/L, diluted in the water where the tadpoles were maintained.	Increased activity of glutathione S-transferase and acetylcholinesterase enzymes, 70% increase in thyroxine levels, significantly decreased average heart rate (indicating cardiotoxicity), reduced swimming speed and general activity.	54
Pregnant rats	Fetuses	Exposure to pyriproxyfen 100 mg/kg, 300 mg/kg, and 500 mg/kg administered through gavage from gestation days 6 to 15.	Skeletal changes suggestive of developmental delay, particularly at the highest doses (300 mg/kg and 500 mg/kg).	55
Adult male rats	Liver microsomes (metabolic studies), hepatocytes (cytotoxicity assays)	*In vitro* incubation of microsomes to pyriproxyfen 1 μM for 1 to 90 minutes.	Enantioselective metabolism in liver microsomes, significant toxicity in hepatocytes resulting in apoptosis and DNA damage.	56
Male Wistar rats	Duodenum and jejunum strips	*In vitro* exposure of the intestinal strips to pyriproxyfen at non-cumulative concentrations varying from 0.032 mM to 100 mM.	Impaired intestinal motoric activity causing dose-dependent muscle relaxation, with duodenum strips showing greater sensitivity than jejunum strips.	57
*Rhamdia quelen* (fish) eggs	Whole-body embryos/larvae	Post-fertilization exposure for 96 hours to pyriproxyfen 1 µg/L and 10 µg/L diluted in water.	Toxicity to embryos causing significantly reduced survival, hatching issues, and deformities.	58
Adult, 8-week-old male rats	Brain (subventricular, zone-derived)	Exposure of cultured neural stem cells for 7 days to pyriproxyfen 3×10^-^¹ mg/L.	Disrupted thyroid hormone signaling, reduced neural stem cell proliferation, increased apoptosis, altered expression of neurodevelopmental gene.	59
*Xenopus tadpoles*	*Xenopus laevis* brains	Exposure for 72 hours to pyriproxyfen 3×10^-^¹ mg/L diluted in water.	Disrupted thyroid hormone signaling, altered expression of neurodevelopmental gene.	59
Zebrafish embryos	Zebrafish embryos/larvae	Post-fertilization exposure for 96 hours to pyriproxyfen 0.16 µg/mL, 0.33 µg/mL, and 1.66 µg/mL diluted in daily renewed water.	Developmental deformities (pericardial edema, scoliosis), oxidative stress, increased reactive oxygen species and lipid peroxidation, inhibited acetylcholinesterase activity at higher concentrations.	60
Adult zebrafish (*Danio rerio*)	Testes and liver	Exposure for 7 days to pyriproxyfen 10^−9^ M diluted in water.	Calcium overload, increased lipid peroxidation, decreased glutathione levels, altered antioxidant defense system, changes in spermatogenesis, increased size and number of spermatogonia cysts.	61
Adult *zebrafish* (*Danio rerio*)	Brain, liver, and gonads (testes and ovaries)	Exposure for 21 days to pyriproxyfen 1 µg/L, 10 µg/L, and 100 µg/L diluted in daily renewed water.	Reproductive endocrine disruption, altered testosterone and estradiol levels, increased vitellogenin levels in males, histopathological damage in gonads (testes and ovaries).	62
Zebrafish embryos(*Danio rerio*)	Whole zebrafish embryos and larvae brains	Immersion, from cell stage 2-4 until post-fertilization day 7, in a water solution with pyriproxyfen at concentrations ranging from 0.005 µg/mL to 1 µg/mL.	Lethal at high doses, with 100% mortality at post-fertilization day 7, no effects on brain development or microcephaly at recommended doses.	63
Adult zebrafish (6-7 months old)	Whole bodies	Immersion for 96 hours in a water solution with pyriproxyfen 0.125 mg/L, 0.675 mg/L, and 1.75 mg/L.	Impaired cognitive parameters (*e.g.*, aversive memory), significantly reduced cortisol levels, no effects on locomotion or anxiety-related behaviors.	37
Zebrafish embryos	Whole embryos	Immersion from 6 to 120 hours post-fertilization in a water solution with pyriproxyfen at concentrations ranging from 0.0064 µM to 64 µM.	Significant mortality and morphological defects (pericardial edema and bent axis) in larvae, particularly at the highest concentrations.	64
Adult mice	Liver, kidneys, testicles	Exposure for 2 years to daily pyriproxyfen 100 ppm (corresponding to a dose of 2 mg/kg of body weight).	Decreased body weight and hepatic effects (*e.g.*, increased relative liver weight and hepatic hypertrophy) were the main results.	65
Lactating goats	Milk, fat (omentum and perirenal), muscles, kidneys, liver	Exposure for 5 consecutive days to pyriproxyfen 10 ppm added to the feeding.	Most of the dose recovered in excrements and contents of the gastrointestinal tract, main residues in milk were pyriproxyfen and its main metabolite (4’-OH-pyriproxyfen sulfate), accumulation of pyriproxyfen in body fat with similar levels in omental and perirenal fat, residual pyriproxyfen also found in muscle, accumulation of 4’-OH-pyriproxyfen sulfate in kidneys.	65
Adult dogs	Behavior	(1-year toxicity study) Exposure to pyriproxyfen 4.7 mg/kg body weight per day (as the No Observed Adverse Effect Level [NOAEL]) added to the feeding.	Manifestations such as diarrhea and salivation, no description of analyzed tissues.	65
Mice (stage of life not specified)	Acetylcholinesterase activity in brain and erythrocytes	(Developmental toxicity study) Exposure for up to 79 weeks to daily pyriproxyfen 50 ppm (equivalent to 6.1 mg/kg body weight) and 250 ppm (equivalent to 32 mg/kg body weight) added to the feeding.	Inhibition of acetylcholinesterase activity in brain and erythrocytes, cholinergic signals and reduced body weight at high concentrations, additional effects included liver changes and increased adrenal weight.	65

Abbreviations: 4’-OH-PPF, pyriproxyfen metabolite; *Msi1*, Musashi-1

In 1999, a collaborative effort between a panel of experts and the WHO was initiated to conduct comprehensive toxicological assessments of pesticide residues in food and in the environment (23). One of the substances under scrutiny was PPF. This document holds significant importance as it established acceptable daily intake values and delved into various biochemical aspects such as absorption, distribution, and excretion, alongside conducting toxicological and genotoxic studies (23,24).

In rats, 96%-98% of PPF is eliminated within 168 hours after administration, primarily through feces, but also through urine and bile (23,24). Residual accumulation corresponds to no more than 0.3% of the dose, with the organ that most bioaccumulates PPF metabolites being the adipose tissue, followed by the liver. Notably, there are no significant differences between sexes regarding excretion rates or organ distribution (23,24). The maximum PPF concentration is reached within 8 hours of administration. Twelve different PPF metabolites have been identified by chromatography, with 4’-OH-pyriproxyfen being the most abundant. In mice subjected to PPF intraperitoneal or intradermal administration, PPF is eliminated more quickly when administered via the intraperitoneal route; as in rats, the distribution of PPF and its metabolites to different organs is more evident in the liver and adipose tissue (23).

In mammals, the effects of acute PPF administration differ depending on the animal model. In rats, administration of PPF at concentrations between 1,000 mg/kg and 5,000 mg/kg delivered through any route (oral, dermal, or aerosol) does not cause death (23) but leads to several effects, *e.g.*, diarrhea and decreased body weight gain, among others. In mice, PPF 2,000 mg/kg causes ataxia, abnormal respiration, and spontaneous movements. In male mice, this dose is sufficient to cause death, while the higher dose of 5,000 mg/kg causes death in both males and females (23). The causes of death are unclear since they are not associated with abnormal organ features, but several manifestations appear after PPF is administered via any route (23,24).

Exposure to PPF has been tested using different *in vivo* models, and its toxic effects in the acute phase and in the short and long term have been analyzed. The different assays showed that the liver is the main organ affected by PPF exposure, increasing plasma lipid concentrations, including cholesterol, among other consequences (23,24).

Due to its central role in metabolizing and detoxifying xenobiotics, the liver is one of the main organs affected by PPF exposure. When a substance like PPF enters the body, the liver is responsible for metabolizing it, often transforming it into metabolites that are easier to excrete (66,67). However, during this process, physiological changes may occur, such as the induction of hepatic enzymes, lipid accumulation, and increased oxidative stress (68,69).

Increases in plasma lipid concentrations, including cholesterol, may be related to PPF's ability to interfere with the regulation of lipid metabolism. Studies indicate that exposure to certain pesticides can cause dysfunction in hepatic metabolic pathways, leading to an imbalance in lipid synthesis and catabolism (70). Additionally, the prolonged activation of metabolic pathways in the liver can result in hepatomegaly and chronic inflammation, contributing to conditions such as fatty liver disease (71,72). Another relevant factor is that the liver tends to be a site of bioaccumulation of PPF metabolites, especially in chronic exposures. This happens because lipophilic compounds, like PPF, have an affinity for fat-rich tissues (73), such as liver and adipose tissues. As PPF and its metabolites accumulate in the liver, the elimination pathways may become overloaded, increasing the organ's susceptibility to structural and functional damage (53).

These mechanisms help explain why the liver is the most affected organ in toxicological studies, highlighting the need to monitor carefully the hepatic effects in prolonged exposures, even at doses considered safe (56).

### Effects of pyriproxyfen exposure on the nervous system

A recent study by Vancamp and cols. showed interesting data suggesting that 4’-OH-pyriproxyfen – the main PPF metabolite – is an active antagonist of thyroid hormones (59). In the study, transgenic tadpoles expressing thyroglobulin (TG) were exposed for 72 hours to 4’-OH-pyriproxyfen in the presence or absence of triiodothyronine (T3) (59). The group exposed to 4’-OH-pyriproxyfen + T3 showed increased TG fluorescence, while the group exposed to 4’-OH-pyriproxyfen alone, even at low doses (10^−7^ nM), showed decreased TG fluorescence. Additionally, tadpoles exposed to 4’-OH-pyriproxyfen at high doses (10^−1^ mg/L and 3×10^−1^ mg/L) presented low mobility, while those exposed to the highest 4’-OH-pyriproxyfen dose (3×10^−1^ mg/L) had decreased head size and disproportionate prosencephalon and mesencephalon dimensions (59). Finally, the study demonstrated that the 4’-OH-pyriproxyfen dose of 3×10^−1^ mg/L decreased the proliferation of neural progenitor cells in neurospheres (59). Thyroid hormones (T3 and thyroxine [T4]) are essential for neurological and cognitive development (74), regulating processes such as neurogenesis, neuronal migration, and the differentiation of nervous system cells (75). As mentioned, 4’-OH-pyriproxyfen acts as an active antagonist of thyroid hormones, decreasing TG fluorescence in transgenic tadpoles, even at low doses. Since brain development is sensitive to thyroid signaling, 4’-OH-pyriproxyfen interference can lead to changes in brain growth, as observed with the reduced head size and disproportionate dimensions of the forebrain and midbrain in tadpoles exposed to this metabolite (59). The decreased proliferation of neural progenitor cells in neurospheres, observed at high 4’-OH-pyriproxyfen doses, suggests that this substance may interfere with the formation and maintenance of cell populations responsible for generating new neurons (76). This effect can result in defective cognitive development and altered brain plasticity. Another objective of the study by Vancamp and cols. was to assess the metabolite's effects on Musashi-1 (MSI1) levels (59). Chavali and cols., in 2017, showed that MSI1 is fundamental for the replication of the Zika virus due to its direct interaction with viral RNA; however, its expression is downregulated in the presence of thyroid hormones (77). The Vancamp and cols. study corroborated this finding by demonstrating that T3 treatment downregulated MSI1 expression compared with no T3 treatment; however, exposure to 4’-OH-pyriproxyfen upregulated MSI1 expression in the presence of T3, demonstrating that this metabolite can alter gene expression in Zika virus (59). The altered MSI1 expression observed suggests that 4´-OH-pyriproxyfen may modify gene expression pathways associated with neural development, in addition to potentially increasing susceptibility to viral infections affecting the brain.

A study investigating the effects of 10 days of PPF 10 mg/L exposure on *Gallus domesticus* embryos revealed a significant increase in apoptotic cells and a notable reduction in brain mass, including the thickness of the cellular layers of the brain and mesencephalon, along with decreased head size (52). The increased apoptosis observed can lead to the loss of essential neuronal cells, impairing cognitive development and brain architecture (78). Although PPF did not affect neuronal and glial differentiation or the cranial ossification process, it proved to be a strong inducer of stress for neurodevelopment, resulting in alterations in the cellular architecture of brain vesicles (52).

Zebrafish male adults treated for 16 hours with PPF at concentrations of 0.001 mg/mL, 0.01 mg/mL, and 0.1 mg/mL exhibited inhibited acetylcholinesterase activity, indicating a nearly concentration-dependent effect on synaptic function (58). Additionally, there was an increase in the generation of oxygen and nitrogen-related species (58), which may lead to neuronal damage (79). Acetylcholinesterase is crucial for synaptic function, breaking down acetylcholine and regulating neurotransmission. Its inhibition can result in synaptic and cognitive dysfunctions, such as learning and memory difficulties (80). Exposure to PPF also increases the generation of reactive oxygen and nitrogen species, leading to an oxidative stress environment that can damage neurons (79,80). Oxidative stress is known to be associated with neurodegenerative diseases and cognitive deficits (81). These findings further strengthen the evidence of a relationship between PPF and microcephaly.

### Effects of pyriproxyfen exposure on the endocrine system

The various applications of PPF resulting in direct or indirect actions on ecosystems and the outcomes from experiments conducted in controlled environments emphasize that the PPF presence cannot be neglected (32,49-52,58,60).

A study investigating the toxic potential of PPF in *Odontophrynus americanus* tadpoles (54) exposed them to the pesticide for 22 days, revealing a reduction in the animals’ heart rate. Also, their swimming pattern appeared compromised, reflected by a reduction in distance traveled, average speed, and overall activity. The PPF concentration of 0.1 mg/L caused a significant increase in serum T4 levels compared with no PPF exposure, suggesting an impact of PPF on thyroid hormones (54). The results also highlighted an increased activity of the enzymes glutathione S-transferase, acetylcholinesterase, and carboxylesterase, indicating a detoxification response (54). A possible explanation for PPF-induced toxicity may be the importance of thyroid hormones during the metamorphic stages in these animals (82).

Notably, PPF can be considered an active antagonist of thyroid hormones due to their structural similarity. The phenolic rings in the structure of both T3 and T4 are crucial for binding to thyroid hormone receptors. Metabolites with similar phenolic groups (*e.g.*, 4’-OH-pyriproxyfen) may compete for these receptors or for specific transport proteins (*e.g.*, transthyretin) with affinity for molecules with phenolic structures.

Moreover, thyroid signaling mechanisms are highly sensitive to chemical interference (83). Compounds that mimic or antagonize these hormones can deregulate thyroid function, affecting various processes, including metabolism, neurological development, and thermoregulation (83). Additionally, PPF may bind to thyroid hormone receptors or interfere with thyroid signaling pathways, acting as an endocrine disruptor. This interference may occur even though the PPF structure is not identical to that of thyroid hormones, as it possesses enough characteristics to occupy the binding sites on receptors and compete with T3 and T4 in metabolic pathways. Thus, this partial structural similarity may be sufficient to explain the antagonistic activity observed in the studies.

**Figure 3:**
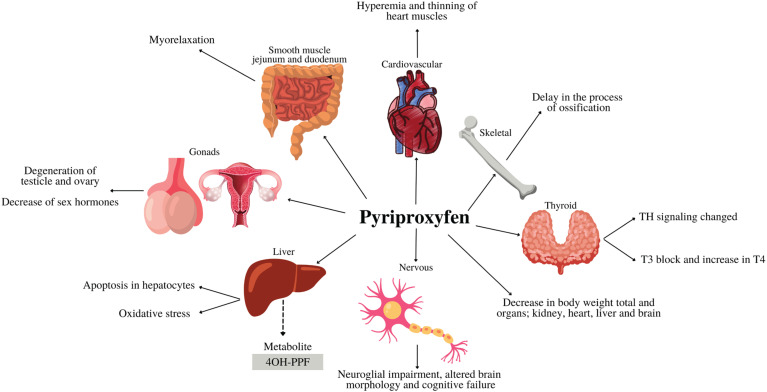
Effects of pyriproxyfen on different systems and organs. The dashed arrow indicates the main pyriproxyfen metabolite. Abbreviations: 4’-OH-PPF, pyriproxyfen metabolite; TH, thyroid hormones; T3, triiodothyronine; T4, thyroxine. Figure created using Canva.

Experiments conducted on zebrafish embryos/larvae (60) between 3 hours post-fertilization to 96 hours, exposed to PPF concentrations of 0.16 μg/mL, 0.33 μg/mL, and 1.66 μg/mL, exhibited interesting morphological effects (Figure 3), including pericardial edema, scoliosis, elongation of the heart, yolk sac edema, hyperemia, and red blood cell accumulation (60).

A study on 30-day-old *Danio rerio* exposed to PPF at concentrations of 15.6 μg/L, 31.2 μg/L, 62.5 μg/L, 125 μg/L, and 250 μg/L suggested that PPF exhibits endocrine-disrupting activity. Both total length and body weight were significantly reduced in the groups treated with 125 μg/L and 250 μg/L compared with the control group. Additionally, growth hormone mRNA expression was lower in these same groups, suggesting inhibition in teleost fish (84).

The effect of PPF as an endocrine disruptor is a key focus of the present review, as several studies have demonstrated that disruptions in the regulation of the endocrine axis – such as thyroid gland dysfunction, receptor abnormalities, hormone transporter protein disorders, and even alterations in hormone levels – can lead to severe diseases (83,85).

From a toxicological context, coexposures must also be considered (86). The importance of coexposure has been demonstrated in a study analyzing animals exposed to PPF along with microcystin (toxins produced by cyanobacteria species). Even low PPF doses led to decreased survival in the first hours and increased deformity rates (*i.e.*, eye and head size, damage to feeding and reproductive structures, abnormality in fin size and curvature, and pericardial edema). These data support the findings of other studies, indicating a possible risk of extinction with these pesticides (37,60,62,63).

Exposure of zebrafish larvae to PPF for 120 hours led to substantial alterations in the treated animals. In the experiments, PPF was used at varying concentrations (0.025 mg/L, 0.125 mg/L, 0.25 mg/L, 1.25 mg/L, 2.5 mg/L, and 10 mg/L), both individually and in combination with diflubenzuron (DFB) to yield different mixtures (34). In concentrations above 2.5 mg/L, PPF-induced teratogenic effects, significantly increasing the number of malformations such as pericardial edema, ocular malformations, and tail defects (34).

Abnormalities in skeletal and cardiac development caused by PPF may also lead to alterations in spinal curvature and elongation of the heart (87). These effects may be indicative of structural-developmental disorders, potentially influenced by PPF's toxicity in early developmental stages (88).

As part of an inflammatory response or due to impaired blood flow regulation, increased blood flow may occur in specific areas (89) and manifest as cellular or tissue damage, possibly caused by oxidative stress or a direct impact of the pesticide.

Several studies have also assessed the effects of PPF on the gonads. In a reproductive toxicity test (50), male rats treated with oral PPF for 28 days showed a significant reduction in body and testicular weight, degenerative changes in testicular tissue, and decreased sperm density. These data indicate that PPF may directly influence male fertility (50). Testicular weight loss can be attributed to cellular damage and degeneration of testicular tissue (90), which can compromise testicular function. These effects are likely caused by oxidative stress or inflammation, resulting in damage to sperm and Sertoli cells, which are essential for spermatogenesis (91). Reduced sperm density is indicative of impaired spermatogenesis and can lead to infertility, which is also a direct consequence of degenerative changes in testicular tissue.

This evidence is further supported by a 2020 study conducted on male and female zebrafish models (62). In the study, the animals were treated for 21 days with PPF at concentrations of 1 μg/L, 10 μg/L, and 100 μg/L, and those treated with higher PPF concentrations developed histopathological alterations in the testes and ovaries. Notably, PPF demonstrates potential estrogenic action and is associated with reproductive changes (62). It also reduces the expression of the *FSHB* and *LHB* genes, suggesting effects on pituitary activity (62). The potential estrogenic action of PPF may interfere with the activity of estrogenic hormones, leading to hormonal imbalances, which explains the problems caused in the development and function of the reproductive organs (92).

Teratogenic effects of PPF have also been previously studied. Prenatal exposure of mice to PPF (55) causes a significant decrease in offspring's body weight. Additionally, histological changes are observed in organs such as the liver, kidney, heart, and brain of exposed offspring (55). Prenatal exposure to PPF results in increased fetal mortality at higher doses and a significant prolongation of the gestational period (55). Oral administration of PPF at doses of 100 mg/kg, 300 mg/kg, or 500 mg/kg daily during the organogenic period in Wistar rats leads to bone alterations suggestive of developmental delay (55).

An *in vitro* study demonstrated that PPF inhibits the reactivity of muscle strips to acetylcholine in a dose-dependent manner, abolishing contraction at high doses. It is likely that PPF affects the smooth muscle motor activity of the duodenum and jejunum through the intestinal cholinergic pathway (57). This suggests that PPF directly interferes with the interaction between acetylcholine and its muscle receptors.

More research is needed to explore the possible toxic effects of PPF on non-target organisms, even at concentrations considered safe by competent bodies. This will provide a deeper understanding of PPF's role and help determine whether the use of PPF is justified for pest control. Several studies reporting effects such as lethargy, organ malformations, and delayed development of the central nervous system suggest that the thyroid axis may be a possible target for endocrine disruption by PPF (93-95). The results of these studies indicate that pregnant women and children should be given particular attention regarding the potential effects of PPF. From an ecotoxicological perspective, the negative effects of PPF on animals such as fish may cause environmental imbalance. Collectively, the evidence presented suggests the potential for new candidates in vector control.

## Data Availability

the figures in this study were created using Canva for Education (www.canva.com).

## References

[1] Almeida LS, Cota ALS, Rodrigues DF (2020). Saneamento, arboviroses e determinantes Ambientais: impactos na saúde urbana. Ciênc Saúde Coletiva.

[2] Morchón R, Bueno-Marí R, Bravo-Barriga D (2023). "Biology, Control and Zoonotic Role of Disease Vectors.". Pathogens.

[3] Asgarian TS, Vatandoost H, Hanafi-Bojd AA, Nikpoor F (2023). Worldwide Status of Insecticide Resistance of Aedes aegypti and Ae. albopictus, Vectors of Arboviruses of Chikungunya, Dengue, Zika and Yellow Fever. J Arthropod Borne Dis.

[4] Paixão ES, Teixeira MG, Rodrigues LC (2018). Zika, chikungunya and dengue: the causes and threats of new and re-emerging arboviral diseases. BMJ Glob Health.

[5] Wilke ABB, Vasquez C, Carvajal A, Medina J, Chase C, Cardenas G (2020). Proliferation of Aedes aegypti in urban environments mediated by the availability of key aquatic habitats. Sci Rep.

[6] Engdahl CS, Tikhe CV, Dimopoulos G (2022). Discovery of novel natural products for mosquito control. Parasit Vectors.

[7] Taracena ML, Bottino-Rojas V, Talyuli OAC, Walter-Nuno AB, Oliveira JHM, Angleró-Rodriguez YI (2018). Regulation of midgut cell proliferation impacts Aedes aegypti susceptibility to dengue virus. PLoS Negl Trop Dis.

[8] Mario M (2022). Modelagem de técnicas de controle populacional do mosquito Aedes aegypti.

[9] Liu N (2015). Insecticide resistance in mosquitoes: impact, mechanisms, and research directions. Annu Rev Entomol.

[10] Pathak VM, Verma VK, Rawat BS, Kaur B, Babu N, Sharma A (2022). Current status of pesticide effects on environment, human health and it's eco-friendly management as bioremediation: A comprehensive review. Front Microbiol.

[11] Ali S, Ullah MI, Sajjad A, Shakeel Q, Hussain A, Inamuddin, Ahamed MI, Lichtfouse E (2021). Sustainable Agriculture Reviews 48: Pesticide Occurrence, Analysis and Remediation Vol. 2 Analysis.

[12] Gad M, Aref SA, Abdelhamid A, Elwassimy MM, Abdel-Raheem SAA (2021). Biologically active organic compounds as insect growth regulators (IGRs): Introduction, mode of action, and some synthetic methods. Curr Chem Lett.

[13] Jindra M, Bittova L (2020). The juvenile hormone receptor as a target of juvenoid "insect growth regulators". Arch Insect Biochem Physiol.

[14] Zhang X, Li S, Liu S (2022). Juvenile hormone studies in Drosophila melanogaster. Front Physiol.

[15] Harris C, Lwetoijera DW, Dongus S, Matowo NS, Lorenz LM, Devine GJ (2013). Sterilising effects of pyriproxyfen on Anopheles arabiensis and its potential use in malaria control. Parasit Vectors.

[16] Wang K, Zhao L, Zhang C, Zhang H, Lian K (2021). Determination of 12 insect growth regulator residues in foods of different matrixes by modified QuEChERS and UPLC-MS/MS. RSC Adv.

[17] Liu N (2015). Insecticide Resistance in Mosquitoes: Impact, Mechanisms, and Research Directions. Annu Rev Entomol.

[18] Parveen K (2021). Dissipation kinetics of pyriproxyfen in chilli (Capsicum annum L.) and soil. Diss.

[19] Arredondo J, Aguirre-Medina JF, Meza-Hernández JS, Cancino J, Díaz-Fleischer F (2024). Accelerating sexual maturation of male Anastrepha ludens (Diptera: Tephritidae) fruit flies by adding two juvenile hormone analogues. Pest Manag Sci.

[20] Perez A (2021). Evaluation of a fitness-disrupting chemical for the control of Aedes albopictus mosquitoes.

[21] Grisales N, Lees RS, Maas J, Morgan JC, Wangrawa DW (2021). Pyriproxyfen-treated bed nets reduce reproductive fitness and longevity of pyrethroid-resistant Anopheles gambiae under laboratory and field conditions. Malar J.

[22] Ahmed TH, Saunders TR, Mullins D, Rahman MZ, Zhu J (2020). The juvenile hormone receptor Methoprene-tolerant is involved in the sterilizing effect of pyriproxyfen on adult Aedes aegypti mosquitoes. bioRxiv.

[23] World Health Organization (2021). World Health Organization, International Programme on Chemical Safety (WHO/PCS/01.5).

[24] World Health Organization (2007). Background document for development of World Health Organization Guidelines for Drinking-water Quality.

[25] Caixeta ES, Silva CF, Santos VS, Olegário de Campos E, Pereira BB (2016). Ecotoxicological assessment of pyriproxyfen under environmentally realistic exposure conditions of integrated vector management for Aedes aegypti control in Brazil. J Toxicol Environ Health A.

[26] Sullivan JJ, Goh KS (2008). Environmental fate and properties of pyriproxyfen. J Pestic Sci.

[27] Suman DS, Wang Y, Faraji A, Williams GM, Williges E, Gaugler R (2018). Seasonal field efficacy of pyriproxyfen autodissemination stations against container-inhabiting mosquito Aedes albopictus under different habitat conditions. Pest Manag Sci.

[28] Martínez LC, Plata-Rueda A, Serrão JE (2021). Effects of Insect Growth Regulators on Mortality, Survival, and Feeding of Euprosterna Elaeasa (Lepidoptera: Limacodidae) Larvae. Agronomy.

[29] Faria Waziry PA, Raja A, Salmon C, Aldana N, Damodar S, Fukushima AR, Mayi BS (2020). Impact of pyriproxyfen on virus behavior: implications for pesticide-induced virulence and mechanism of transmission. Virol J.

[30] Li H, Zhong Q, Wang M, Luo F, Wang X, Zhou L (2022). Residue degradation, transfer and risk assessment of pyriproxyfen and its metabolites from tea garden to cup by ultra performance liquid chromatography tandem mass spectrometry. J Sci Food Agric.

[31] dos Santos RPA, Cruz WDF, Magalhães KF, Araújo DM, Medeiros MC, Martinez-Huitle CA (2020). Electrochemical degradation of a commercial formulation of the insecticide pyriproxyfen using boron-doped diamond anode. J Electrochem Soc.

[32] Devillers J (2020). Fate of pyriproxyfen in soils and plants. Toxics.

[33] Ngesom Mohd, Mohiddin Ahmad (2021). "Evaluating the potential of pyriproxyfen dissemination using mosquito home system against Aedes albopictus at a dengue hotspot area. Sains Malaysiana.

[34] Teixeira JRS, de Souza AM, Macedo-Sampaio JV, Menezes FP, Pereira BF, de Medeiros SRB (2024). Embryotoxic effects of pesticides in zebrafish (Danio rerio): diflubenzuron, pyriproxyfen, and its mixtures. Toxics.

[35] Maharajan K, Muthulakshmi S, Karthik C, Nataraj B, Nambirajan K, Hemalatha D (2020). Pyriproxyfen induced impairment of reproductive endocrine homeostasis and gonadal histopathology in zebrafish (Danio rerio) by altered expression of hypothalamus-pituitary-gonadal (HPG) axis genes. Sci Total Environ.

[36] Parens R, Nijhout HF, Morales A, Xavier Costa F, Bar-Yam Y (2017). A possible link between pyriproxyfen and microcephaly. PLoS Curr.

[37] Gusso D, Reolon GK, Gonzalez JB, Altenhofen S, Kist LW, Bogo MR (2020). Pyriproxyfen Exposure Impairs Cognitive Parameters and Alters Cortisol Levels in Zebrafish. Front Behav Neurosci.

[38] Lajmanovich RC, Peltzer PM, Martinuzzi CS, Attademo AM, Bassó A, Colussi CL (2019). Insecticide pyriproxyfen (Dragón^®^) damage biotransformation, thyroid hormones, heart rate, and swimming performance of Odontophrynus americanus tadpoles. Chemosphere.

[39] Ose K, Miyamoto M, Fujisawa T, Katagi T (2017). Bioconcentration and metabolism of pyriproxyfen in tadpoles of African clawed frogs, Xenopus laevis. J Agric Food Chem.

[40] Salesa B, Torres-Gavilá J, Sancho E, Ferrando MD (2023). Multigenerational effects of the insecticide Pyriproxyfen and recovery in Daphnia magna. Sci Total Environ.

[41] Salesa B, Torres-Gavilá J, Ferrando-Rodrigo MD, Sancho E (2024). Pyriproxyfen Contamination in Daphnia magna: Identifying Early Warning Biomarkers. J Xenobiot.

[42] Reis Vilma Nota técnica sobre microcefalia e doenças relacionadas ao Aedes aegypti: os perigos das abordagens com larvicidas e nebulizações químicas – fumacê.

[43] Mlakar J, Korva M, Tul N, Popović M, Poljšak-Prijatelj M, Mraz J (2016). Zika virus associated with microcephaly. N Engl J Med.

[44] Yuan L, Huang XY, Liu ZY, Zhang F, Zhu XL, Yu JY (2017). A single mutation in the prM protein of Zika virus contributes to fetal microcephaly. Science.

[45] Devillers J, Devillers H (2020). Lethal and Sublethal Effects of Pyriproxyfen on Apis and Non-Apis Bees. Toxics.

[46] Shahid A, Zaidi SD, Akbar H, Saeed S (2019). An investigation on some toxic effects of pyriproxyfen in adult male mice. Iran J Basic Med Sci.

[47] Devillers J (2020). Fate of Pyriproxyfen in Soils and Plants. Toxics.

[48] Kumari P, Duhan A, Rani N, Tomar D (2021). Ultimate Fate and Toxicological Consequences of Insecticide Pyriproxyfen and Its Metabolites in Soil Ecosystem. Environ Adv.

[49] Spirhanzlova P, Le Mével S, Wejaphikul K, Mughal B, Gothié JD, Sébillot A (2018). The pyriproxyfen metabolite 4'OH- pyriproxyfen disrupts thyroid hormone signaling and enhances Musashi-1 levels in neuroprogenitors. bioRxiv.

[50] Shahid A, Saher M (2020). Repeated Exposure of Pyriproxyfen to Pregnant Female Mice Causes Developmental Abnormalities in Prenatal Pups. Environ Sci Pollut Res Int.

[51] Scudeler EL, Carvalho SF, Garcia ASG, Santorum M, Padovani CR, Santos DCD (2022). Midgut and fat body: Multisystemic action of pyriproxyfen on non-target organism Ceraeochrysa claveri (Neuroptera: Chrysopidae). Environ Pollut.

[52] Luckmann MR, de Melo MS, Spricigo MC, da Silva NM, Nazari EM (2021). Pyriproxyfen exposure induces DNA damage, cell proliferation impairments and apoptosis in the brain vesicles layers of chicken embryos. Toxicology.

[53] Nimet J, Leite NF, Paulin AF, Margarido VP, Moresco RM (2021). Use of High-Performance Liquid Chromatography-Mass Spectrometry of Adipose Tissue for Detection of Bioaccumulation of Pyriproxyfen in Adults of Lithobates catesbeianus. Bull Environ Contam Toxicol.

[54] Schlenk D, Almeida EA, Almeida EA, Freitas JS (2023). Toxicology of Amphibian Tadpoles.

[55] Sartori AS, Hollenbach CB, Jardim LH, Silva P, Mello FB, Mello JRB (2020). Avaliação da toxicidade pré-natal: estudo de teratogenicidade do inseticida piriproxifeno em ratos Wistar. Arq Bras Med Vet Zootec.

[56] Liu H, Li P, Wang P, Liu D, Zhou Z (2020). Toxicity risk assessment of pyriproxyfen and metabolites in the rat liver: A vitro study. J Hazard Mater.

[57] Chłopecka M, Mendel M, Dziekan N, Karlik W (2018). The Effect of Pyriproxyfen on the Motoric Activity of Rat Intestine - In Vitro Study. Environ Pollut.

[58] Azevedo RDS, Falcão KVG, Assis CRD, Martins RMG, Araújo MC, Yogui GT (2021). Effects of pyriproxyfen on zebrafish brain mitochondria and acetylcholinesterase. Chemosphere.

[59] Vancamp P, Spirhanzlova P, Sébillot A, Butruille L, Gothié JD, Le Mével S (2021). The Pyriproxyfen Metabolite, 4′–OH–PPF, Disrupts Thyroid Hormone Signaling in Neural Stem Cells, Modifying Neurodevelopmental Genes Affected by ZIKA Virus Infection. Environ Pollut.

[60] Maharajan K, Muthulakshmi S, Karthik C, Nataraj B, Nambirajan K, Hemalatha D (2020). Pyriproxyfen induced impairment of reproductive endocrine homeostasis and gonadal histopathology in zebrafish (Danio rerio) by altered expression of hypothalamus-pituitary-gonadal (HPG) axis genes. Sci Total Environ.

[61] Staldoni de Oliveira V, Gomes Castro AJ, Marins K, Bittencourt Mendes AK, Araújo Leite GA, Zamoner A (2021). Pyriproxyfen induces intracellular calcium overload and alters antioxidant defenses in Danio rerio testis that may influence ongoing spermatogenesis. Environ Pollut.

[62] Maharajan K, Muthulakshmi S, Nataraj B, Ramesh M, Kadirvelu K (2018). Toxicity assessment of pyriproxyfen in vertebrate model zebrafish embryos (Danio rerio): a multi biomarker study. Aquat Toxicol.

[63] Dzieciolowska S, Larroque AL, Kranjec EA, Drapeau P, Samarut E (2017). The larvicide pyriproxyfen blamed during the Zika virus outbreak does not cause microcephaly in zebrafish embryos. Sci Rep.

[64] Truong L, Gonnerman G, Simonich MT, Tanguay RL (2016). Assessment of the developmental and neurotoxicity of the mosquito control larvicide, pyriproxyfen, using embryonic zebrafish. Environ Pollut.

[65] PCS (1999). FAO Panel of Experts on Pesticide Residues in Food and the Environment Toxicological Evaluations.

[66] Stanley LA (2024). Metabolismo de fármacos, Farmacognosia.

[67] Shanu-Wilson J, Evans L, Wrigley S, Steele J, Atherton J, Boer J (2020). Biotransformation: impact and application of metabolism in drug discovery. ACS Med Chem Lett.

[68] Villanueva-Paz M, Morán L, López-Alcántara N, Freixo C, Andrade RJ, Lucena MI (2021). Oxidative stress in drug-induced liver injury (DILI): from mechanisms to biomarkers for use in clinical practice. Antioxidants (Basel).

[69] Arroyave-Ospina JC, Wu Z, Geng Y, Moshage H (2021). Role of oxidative stress in the pathogenesis of non-alcoholic fatty liver disease: implications for prevention and therapy. Antioxidants (Basel).

[70] He B, Ni Y, Jin Y, Fu Z (2020). Pesticides-induced energy metabolic disorders. Sci Total Environ.

[71] Cano R, Pérez JL, Dávila LA, Ortega Á, Gómez Y, Valero-Cedeño NJ (2021). Role of endocrine-disrupting chemicals in the pathogenesis of non-alcoholic fatty liver disease: a comprehensive review. Int J Mol Sci.

[72] Rajak S, Raza S, Tewari A, Sinha RA (2022). Environmental toxicants and NAFLD: a neglected yet significant relationship. Dig Dis Sci.

[73] Li X, Zhang C, Wang K, Lehmler HJ (2020). Fatty liver and impaired hepatic metabolism alter the congener-specific distribution of polychlorinated biphenyls (PCBs) in mice with a liver-specific deletion of cytochrome P450 reductase. Environ Pollut.

[74] Giannocco G, Kizys MML, Maciel RM, de Souza JS (2021). Thyroid hormone, gene expression, and central nervous system: where we are. Semin Cell Dev Biol.

[75] Montero-Pedrazuela A, Grijota-Martínez C, Ausó E, Bárez-López S, Guadaño-Ferraz A, Martin CR, Preedy VR, Rajendram R (2021). Diagnosis, Management and Modeling of Neurodevelopmental Disorders.

[76] da Silva Siqueira L, Majolo F, da Silva APB, da Costa JC, Marinowic DR (2021). Neurospheres: a potential in vitro model for the study of central nervous system disorders. Mol Biol Rep.

[77] Chavali PL, Stojic L, Meredith LW, Joseph N, Nahorski MS, Sanford TJ (2017). Neurodevelopmental Protein Musashi-1 Interacts with the Zika Genome and Promotes Viral Replication. Science.

[78] Hess JL, Radonjić NV, Patak J, Glatt SJ, Faraone SV (2021). Autophagy, apoptosis, and neurodevelopmental genes might underlie selective brain region vulnerability in attention-deficit/hyperactivity disorder. Mol Psychiatry.

[79] Yan X, Liu Y, Zhang X, Zhang Q, Liu Y, Guo Y (2024). Atmospheric pressure plasma preconditioning reduces oxygen and glucose deprivation induced human neuronal SH-SY5Y cells apoptosis by activating protective autophagy and ROS/AMPK/mTOR pathway. Cell Signal.

[80] Huang Q, Liao C, Ge F, Ao J, Liu T (2022). Acetylcholine bidirectionally regulates learning and memory. J Neurorestoratol.

[81] Teleanu DM, Niculescu AG, Lungu II, Radu CI, Vladâcenco O, Roza E (2022). An overview of oxidative stress, neuroinflammation, and neurodegenerative diseases. Int J Mol Sci.

[82] Ruthsatz K, Dausmann KH, Reinhardt S, Robinson T, Sabatino NM, Peck MA (2020). Post-metamorphic carry-over effects of altered thyroid hormone level and developmental temperature: physiological plasticity and body condition at two life stages in Rana temporaria. J Comp Physiol B.

[83] O'Shaughnessy KL, Gilbert ME (2020). Thyroid disrupting chemicals and developmental neurotoxicity - New tools and approaches to evaluate hormone action. Mol Cell Endocrinol.

[84] Horie Y, Mitsunaga K, Yap CK (2023). Pyriproxyfen Influences Growth as Well as Thyroid Hormone-Related and Gh/Igf-1 Gene Expression during the Early Life Stage of Zebrafish (Danio Rerio). Comp Biochem Physiol C Toxicol Pharmacol.

[85] Lagadic L, Coady KK, Körner O, Miller TJ, Mingo V, Salinas ER (2024). Endocrine disruption assessment in aquatic vertebrates - Identification of substance-induced thyroid-mediated effect patterns. Environ Int.

[86] Tralau T, Oelgeschläger M, Kugler J, Bloch D, Braeuning A, Burgdorf T (2021). A prospective whole-mixture approach to assess risk of the food and chemical exposome. Nat Food.

[87] Xie H, Kang Y, Liu J, Huang M, Dai Z, Shi J (2023). Ependymal polarity defects coupled with disorganized ciliary beating drive abnormal cerebrospinal fluid flow and spine curvature in zebrafish. PLoS Biol.

[88] Sun Y, Cao Y, Tong L, Tao F, Wang X, Wu H (2020). Exposure to prothioconazole induces developmental toxicity and cardiovascular effects on zebrafish embryo. Chemosphere.

[89] Kugler E, Snodgrass R, Bowley G, Plant K, Serbanovic-Canic J, Hamilton N (2021). The effect of absent blood flow on the zebrafish cerebral and trunk vasculature. Vasc Biol.

[90] Hussain AlDulaimi L (2024). Effect of Oxidative Stress on Histological and Immunohistochemical Changes in Testes of Albino Mice. Iran J Vet Med.

[91] Bashghareh A, Rastegar T, Modarresi P, Kazemzadeh S, Salem M, Hedayatpour A (2024). Recovering spermatogenesis by protected cryopreservation using metformin and transplanting spermatogonial stem cells into testis in an azoospermia mouse model. Biopreserv Biobank.

[92] Amir S, Shah STA, Mamoulakis C, Docea AO, Kalantzi OI, Zachariou A (2021). Endocrine disruptors acting on estrogen and androgen pathways cause reproductive disorders through multiple mechanisms: a review. Int J Environ Res Public Health.

[93] Thambirajah AA, Wade MG, Verreault J, Buisine N, Alves VA, Langlois VS (2022). Disruption by stealth-Interference of endocrine disrupting chemicals on hormonal crosstalk with thyroid axis function in humans and other animals. Environ Res.

[94] Cediel-Ulloa A, Lupu DL, Johansson Y, Hinojosa M, Özel F, Rüegg J (2022). Impact of endocrine disrupting chemicals on neurodevelopment: the need for better testing strategies for endocrine disruption-induced developmental neurotoxicity. Expert Rev Endocrinol Metab.

[95] Baksi S, Pradhan A (2021). Thyroid hormone: sex-dependent role in nervous system regulation and disease. Biol Sex Differ.

